# Multi-Sensor Image and Range-Based Techniques for the Geometric Documentation and the Photorealistic 3D Modeling of Complex Architectural Monuments

**DOI:** 10.3390/s24092671

**Published:** 2024-04-23

**Authors:** Alexandra Tsiachta, Panagiotis Argyrou, Ioannis Tsougas, Maria Kladou, Panagiotis Ravanidis, Dimitris Kaimaris, Charalampos Georgiadis, Olga Georgoula, Petros Patias

**Affiliations:** 1 School of Rural and Surveying Engineering, Aristotle University of Thessaloniki, 54124 Thessaloniki, Greece; argyroupa@arch.auth.gr (P.A.); kladoumaria@arch.auth.gr (M.K.); harrisg@auth.gr (C.G.); olge@auth.gr (O.G.); patias@auth.gr (P.P.); 2 Department of Planning and Regional Development, University of Thessaly, 38334 Volos, Greece; itsougas@arch.auth.gr; 3 School of Spatial Planning and Development, Aristotle University of Thessaloniki, 54124 Thessaloniki, Greece; ravanidp@arch.auth.gr (P.R.); kaimaris@auth.gr (D.K.)

**Keywords:** geometric documentation, 3D modeling, UAV, photogrammetry, terrestrial photogrammetry, laser scanning, cultural heritage

## Abstract

The selection of the optimal methodology for the 3D geometric documentation of cultural heritage is a subject of high concern in contemporary scientific research. As a matter of fact, it requires a multi-source data acquisition process and the fusion of datasets from different sensors. This paper aims to demonstrate the workflow for the proper implementation and integration of geodetic, photogrammetric and laser scanning techniques so that high-quality photorealistic 3D models and other documentation products can be generated for a complicated, large-dimensional architectural monument and its surroundings. As a case study, we present the monitoring of the Mehmet Bey Mosque, which is a landmark in the city of Serres and a significant remaining sample of the Ottoman architecture in Greece. The surveying campaign was conducted in the context of the 2022–2023 annual workshop of the Interdepartmental Program of Postgraduate Studies “Protection Conservation and Restoration of Cultural Monuments” of the Aristotle University of Thessaloniki, and it served as a geometric background for interdisciplinary cooperation and decision-making on the monument restoration process. The results of our study encourage the fusion of terrestrial laser scanning and photogrammetric datasets for the 3D modeling of the mosque, as they supplement each other as regards geometry and texture.

## 1. Introduction

Cultural heritage documentation is an interdisciplinary process that includes a wide range of activities, from data acquisition and processing to visualization and information management [1] (p. 1). Computer vision techniques have allowed the creation of virtual reality environments with the three-dimensional modeling of cultural monuments, collections of historical objects or panoramas of archaeological sites. The main purposes of these interactive visualization techniques include the documentation in case of damage, the creation of virtual tours and the support of educational processes or restoration projects [2] (p. 99).

The protection of architectural monuments starts with monitoring and documenting their existing state so that further deterioration can be prevented with carefully planned intervention strategies. This process may also facilitate the partial reconstruction of a building according to its initial phase or the reinforcement of its structural stability [3] (p. 2818). Geometric documentation can be considered as a fundamental stage in the process of analysis and diagnosis of the building’s conservation state, as it creates a geometric basis for the reference of every other product and serves the aim of an architectural–historical integrated documentation of the monument [4] (p. 1). The study of all the experts involved is based on information about the current state of the object, so the geometric documentation must be performed in such a way that it supports the whole project [5] (p. 740).

Three-dimensional geometric documentation can be described as the recording of the position, size, shape and the existing state of a monument or an archaeological site in a specific moment of time [6] (p. 699). It is based on a series of measurements, imagery and point cloud data collection, from which visual products with metric properties (i.e., orthophotos, 3D models, vector architectural drawings, etc.) may be produced. The drawings actually present the orthogonal projections of the monument on selected horizontal or vertical planes. The scale and accuracy of the products should be carefully defined before the conduct of the field campaign [7] (pp. 181–182).

Many applications, such as digital documentation and mapping, require high geometric accuracy, photorealism and detailed visualization of the object, as well as the automation and the low cost of the modeling technique. Therefore, selecting the most appropriate technique to meet all the expectations can be a challenge [8] (p. 269). The surveying methods may range from the conventional simple topometric methods, for partially or totally uncontrolled surveys, to innovative surveying and photogrammetric ones for completely controlled surveys. The topometric methods can be used only when the monument is simple with small dimensions and an uncontrolled survey is adequate, or as a small completion for fully controlled surveys [9] (p. 33). The different sizes, shapes, locations and environmental conditions of the objects require data acquisition by means of aerial and terrestrial digital photogrammetric methods (image-based) and laser scanning (range-based) [10] (p. 37). The photogrammetric method is used for the recording of complex objects with a multitude of details (i.e., facades of historical buildings, floor plans, etc.), when access to the monument is limited or when direct contact with the object is prohibited [11] (pp. 45–46). The range-based techniques capture the 3D geometric information of an object directly with the use of laser beam radiation and they can be applied in mapping, recording as-built infrastructures, 3D reconstructions, vegetation, city modeling and other scientific fields, such as forestry, hydrology and geophysics [12] (p. 79).

On the one hand, image-based modeling techniques require some experience in the procedures of data acquisition and processing, with part of it still being manual. On the other hand, the range-based method needs a large budget and much processing time and can prove unpractical in certain projects. Another disadvantage that most of the active sensors present is the lack or the low quality of texture information, which is usually acquired with a separate digital camera and registered afterward onto the laser scanning data for a complete texture mapping [13] (p. 199). The integration of the two datasets is usually an approach that can lead to optimal results in complex structures or sites [14] (p. 455).

As a matter of fact, selecting the most suitable methodology is of crucial importance, because it determines the quality, the time and the cost of a survey. Several factors must be taken into consideration in this initial assessment, such as the geometric and material characteristics of the monument (minimum size of detail, presence of any translucent, reflective or very dark surfaces), the accessibility of the object, the purpose of the campaign and the characteristics of the surveying instruments (accuracy and uncertainty of measurement, geometric and radiometric resolution, etc.) [15] (p. 1.2). The monuments usually present multi-scalar geometrical complexities and variations that do not allow the definition of standard methodologies for every case, so the optimization of the whole pipeline is considered as a fundamental task [16] (p. 153).

This paper aims to show how three-dimensional data acquired with survey, image and range-based techniques can result in the optimal 3D modeling of a complex case study with high geometric accuracy and photorealism so that restoration interventions or even the reconstruction of the monument can be supported [17] (p. 306). The main object of the campaign was a large-dimension mosque with complicated geometry, intense elevations and dense vegetation nearby, factors which caused some difficulties during the data acquisition and processing. In addition, there was no metric information about the monument and the site from other surveys. The topographic survey was conducted with a geodetic station and a GNSS geodetic receiver, while the monument was recorded with close-range aerial and terrestrial photogrammetry methods, as well as with the use of a terrestrial laser scanner. The 3D model was generated both through photogrammetric processing and through the fusion of laser scans with photogrammetric data so that we could carry out a comparative evaluation of the results.

**Figure 1:**
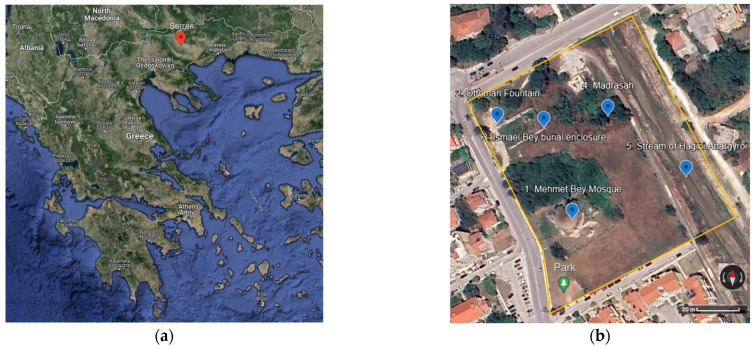
Location on Google Maps: (**a**) the city of Serres; (**b**) the Mehmet Bey complex with location marks on (1) the Mehmet Bey Mosque, (2) the Ottoman Fountain, (3) the Ismael Bey burial enclosure, (4) the Madrasah, (5) the stream of Hagioi Anargyroi.

**Figure 2:**
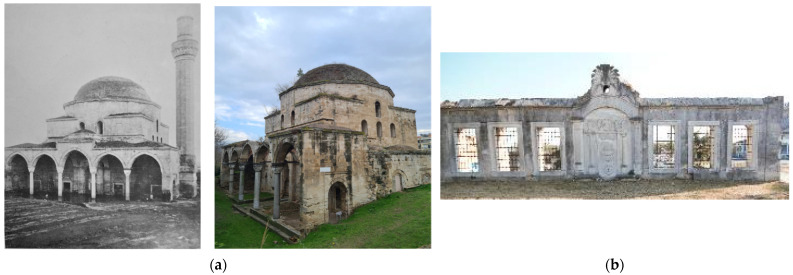
The Mehmet Bey complex: (**a**) the main (north-western) facade of the mosque in 1926 and in 2023 with soil deposition; (**b**) the Ottoman Fountain.

**Figure 3:**
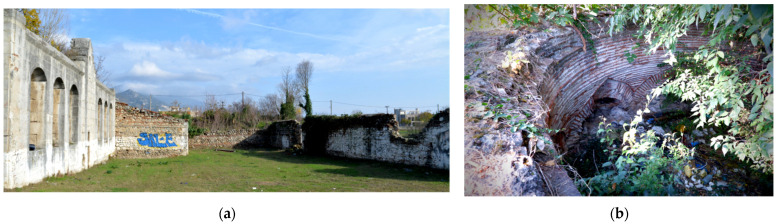
The Mehmet Bey complex: (**a**) the Ismael Bey burial enclosure; (**b**) the Madrasah.

Our monument, the Mehmet Bey Mosque—also known as Ahmet Pasha Mosque or “Hagia Sophia” (41°05′30.4″ N 23°33′34.3″ E) because of its resemblance to the Hagia Sophia Church of Istanbul in shape—is located in the south-eastern outskirts of the city of Serres, Greece (Figure 1a), and it belongs to an Ottoman historical complex (Figure 1b) together with a fountain (Figure 2b), the burial enclosure of Ismael Bey (Figure 3a) and the ruins of a Madrasah (Figure 3b). According to an Arabic inscription above the main entrance, it was built in 1492–1493 by order of Mehmet Bey, son of the Grand Vizier, Gedik Ahmet Pasha. Its dimensions are 29.58 m × 30 m, and the front portico of the main facade measures 5.93 × 30 m (Figure 2a) [18] (pp. 87–89).

It is worth mentioning that this mosque is the oldest and the largest of the three surviving in Serres and a particularly important sample of the early Ottoman architecture. The surviving evidence of Ottoman architecture in Greece, and especially the few Ottoman mosques remaining in the cities of Macedonia, date from the second half of the 14th century until the end of the 19th century, and they are a significant piece of historical and cultural heritage. Some of them have been fully restored, rebuilt or repurposed, and a few have been preserved or partially restored, while others remain in a state of ruins [19] (p. 85).

The Mehmet Bey Mosque was listed as a monument in 1936 [20] (p. 234), but the minaret had already been demolished and the territory had changed dramatically due to the successive floodings of the nearby stream of Hagioi Anargyroi (Figure 1b) from the second half of the 19th until the 20th century. Some restoration interventions have been carried out in the early 2000s, but it is currently closed and neglected, with part of the front portico being collapsed and many problems in the interior [21] (pp. 300–306).

## 2. Methodology

The digitization of the Mehmet Bey complex was implemented through land surveying, close-range photogrammetry and terrestrial laser scanning techniques. The 3D modeling of the main building, the Mehmet Bey Mosque, was conducted both through the fusion of aerial and terrestrial imagery and the fusion of imagery with laser scans with the use of control points. This campaign resulted in the production of a survey plan, an orthophotomosaic, a 3D model, a DTM, a DSM and longitudinal sections of the building block, as well as 3D models, facade orthophotos, orthorectified horizontal and vertical sections and architectural plans of the monuments.

**Figure 4:**
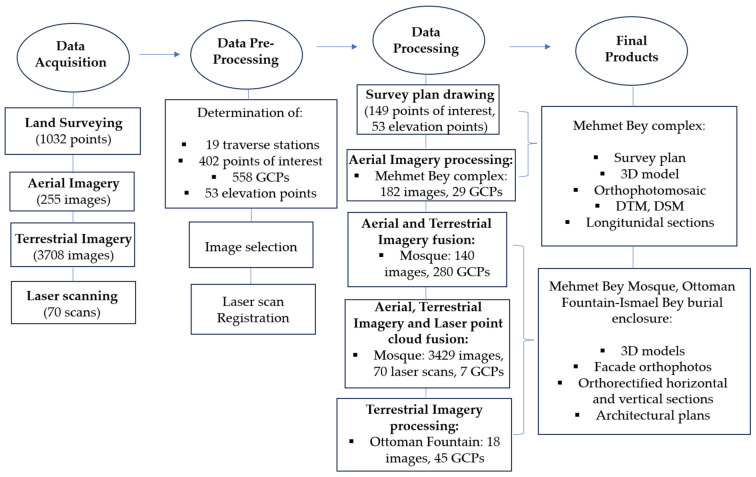
Block diagram of the research procedure.

A block diagram of the workflow regarding the data collection methods, the processing and the generation of products is presented in Figure 4. The total number of acquired points and images is greater than those that were used during the processing, as there has been a selection of the most appropriate. The methodology is described in detail in the following sections.

### 2.1. Data Collection

#### 2.1.1. Land Surveying

The topographic survey of the monumental complex was carried out with the use of a geodetic station, following the establishment of a polygonometric network for the determination of points of interest, ground control points (GCPs), as well as control and check points (CPs) on the facades and in the mosque’s interior surfaces for photogrammetric processing and accuracy evaluation. The level of detail was specified by the drawing scale, which is 1:500 for the survey plan and 1:50 for the plans of the facades, the horizontal and the vertical sections. The graphic accuracy of the plans is estimated at 0.3 mm × 500 = 0.15 m and 0.3 × 50 = 0.015 m, respectively, considering the eye discrimination threshold as 0.3 mm.

**Figure 5:**
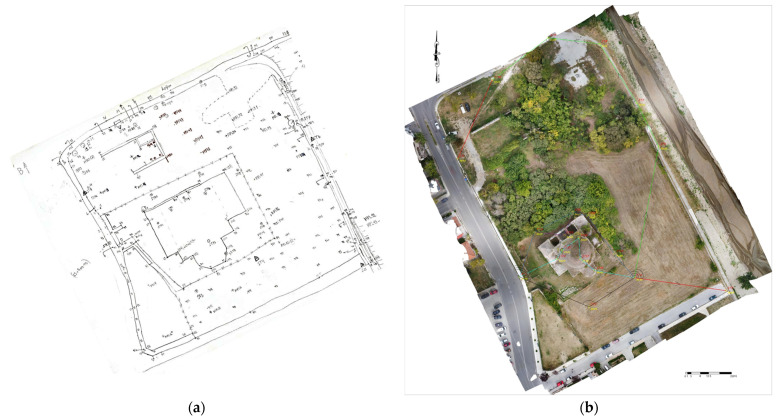
Fieldwork: (**a**) field sketch; (**b**) depiction of the traverses with different colored lines.

The data acquisition process began with the field recognition, the creation of a field sketch (Figure 5a), and the implementation of the polygonometric network’s vertices with marking pegs and wooden stakes on the ground. For the identification of points of interest and control points on the monument internally and externally, as well as GCPs in the entire block, five open, fully dependent and oriented polygon traverses with a total of 19 vertices were created (Figure 5b). Three of the traverses were placed around the monument and one inside so that control point measurements on every surface could be conducted.

**Figure 6:**
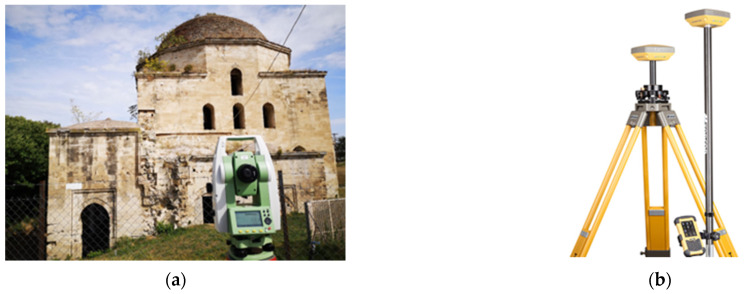
Surveying equipment: (**a**) Leica TS06 Plus R500 Reflectorless total station in the field; (**b**) Topcon Hiper SR GNSS receiver [24].

The survey was carried out using the geodetic station TS06 Plus R500 Reflectorless (Figure 6a), manufactured by Leica Geosystems in Heerbrugg, Switzerland, which gives linear measurement accuracy of 1.5 mm + 2 ppm with a prism and of 2 mm + 2 ppm without a prism [22]. The total station, as well as the accompanying equipment (tripod, tape, prism rod, prism), was provided by the Department of Rural and Surveying Engineering of the Aristotle University of Thessaloniki. In total, 1032 points were identified for the topographic and photogrammetric survey of the mosque, the fountain and the entire block.

The horizontal and vertical networks were referenced on the Greek Geodetic Reference System (GGRS’87), with the dependency points being determined through Static Positioning using a GNSS receiver. The static measurements were carried out on four of the vertices with an accuracy of 3 mm + 0.5 ppm horizontally and 5 mm + 0.5 ppm vertically with the Topcon Hiper SR GNSS receiver mounted on a tripod (Figure 6b) [23]. The receiver was manufactured by Topcon in Tokyo, Japan and it was provided by the School of Spatial Planning and Development of the Aristotle University of Thessaloniki. The measurements at each point lasted for 20–25 min, and their coordinates were approximately confirmed using the RTK method (Real-Time Kinematic Positioning) with the receiver mounted on a spear and the measurements lasting for a few seconds.

#### 2.1.2. Aerial Photogrammetry

**Figure 7:**
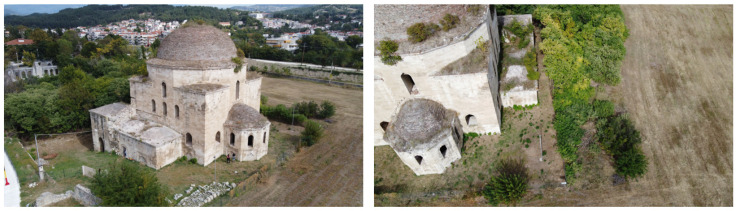
UAV imagery around the mosque.

The aerial photogrammetric survey of the building block and the monument was carried out with the flight of an unmanned aerial vehicle (UAV) and the simultaneous measurement of GCPs. The aerial photography was conducted with the DJI Mavic Air 2 drone quadcopter UAV with a 48 MP camera [25] (Table 1), manufactured by DJI in Shenzhen, China, with which 255 images were acquired (Figure 7). A flight around the mosque, at a height of approximately 18 m above the ground, provided us with a series of oblique overlapping images. A second series of overlapping images was acquired with the camera in a near-vertical position at a height of 43 m so that the wider area could be captured for the production of digital surface models and the orthophotomosaic of the block.

The GCPs were established with special targets (tennis balls) at scattered points that were visible during the drone flight so that they would be distinguishable in the aerial imagery. The area was covered using 29 GCPs. Their location was determined using a geodetic station, and they were marked with code numbers on the field sketch of the topographic survey. With this procedure, we obtained data for the entire block, even for inaccessible points, such as the upper parts of the monument and the stream bed.

#### 2.1.3. Terrestrial Photogrammetry

**Figure 8:**
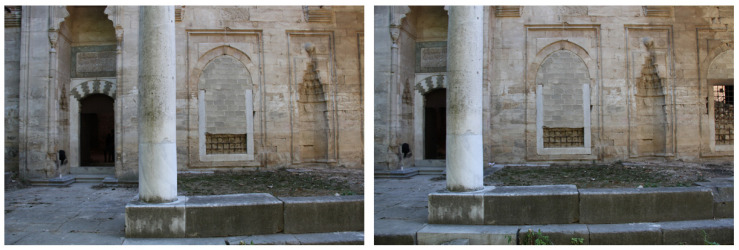
Terrestrial images of the northern facade with high horizontal overlap.

The terrestrial photogrammetric survey was conducted with a Canon EOS 7D DSLR camera (Table 2) [27], manufactured by Canon in Ōita, Japan, aiming for a horizontal overlap of images in the order of 80% (Figure 8) and a vertical overlap between two elevation zones in the order of 60%. The fact is that insufficient overlap in the photos used for photogrammetric reconstruction will result in a 3D model that lacks detail or even contains holes [28] (p. 2). The distance from the object in each series of image acquisition was kept as constant as possible so that errors due to different image scales would be avoided in the process of the bundle adjustment. The height of the mosque necessitated photographing the western and southern facades from a greater distance than the others (approx. 10 m) so that the upper zones of the mosque could be captured. On the eastern and northern faces, however, which were surrounded by nearby fencing and dense vegetation, it was necessary to take oblique photographs from a shorter distance (less than 5 m). Some images were acquired from accessible open window frames of the mosque at a height of 10 m for the recording of the floor.

In total, we collected 3500 terrestrial images for the exterior and the interior of the mosque and 208 for the fountain and the burial enclosure of Ismael Bey. An attempt was made to photograph under uniform lighting conditions to avoid recording solar reflections. Generally, much attention should be paid to the illumination conditions and to the selection of the most appropriate resolution with respect to distance and the level of detail [29] (p. 354). A part of the eastern internal lateral space of the mosque had poor lighting conditions, and some of the images were inappropriate for photogrammetric processing.

**Table 2:** Canon EOS 7D DSLR camera parameters [30].

Model Name	Canon EOS 7D DSLR Camera
Sensor	18MP APS-C CMOS
Image Stabilization	None
Max Shutter Speed	1/8000 s
Min Shutter Speed	30 s
Continuous shooting	8 frames per second
Form Factor	Mid-size SLR
AF system	19 points
Wide-Angle Zoom Lens	Sigma 10–20 mm; F3.5; AF

**Figure 9:**
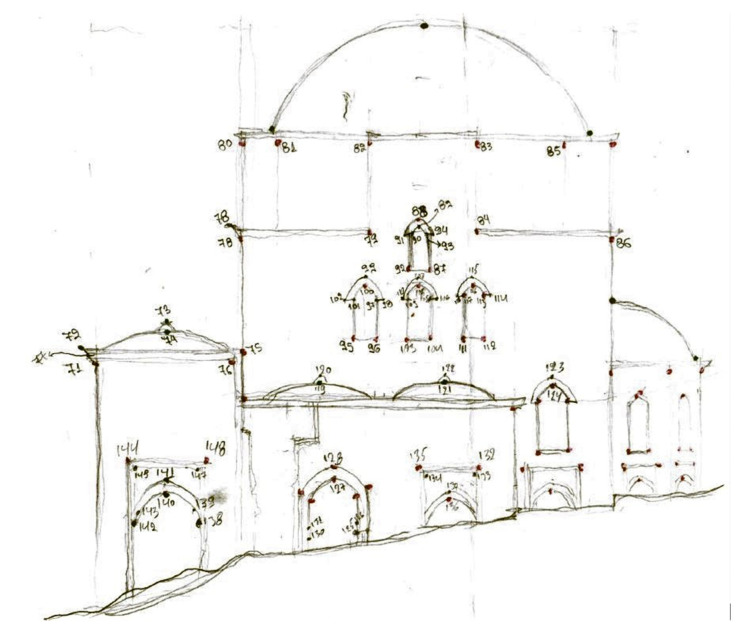
Sketch of the western facade with marked control points.

Regarding the control point establishment, characteristic points of the facades were selected in a uniform distribution. These points were aimed with a geodetic station placed on traverse stations nearby and marked on the sketches of the facades with code numbers (Figure 9). The measurements were implemented by direct aiming at the points since the geodetic station used allows the electronic recording of distances without the use of a prism.

#### 2.1.4. Terrestrial Laser Scanning (TLS)

In recent years, the use of 3D laser scanners in cultural heritage surveying has been a great technological advance [31] (p. 1). Documentation with this method facilitates the creation of real-scale dense point clouds, thus supporting architectural and historical research and allowing the conduction of multi-temporal comparisons [32] (p. 115). Terrestrial 3D laser scanners are significant tools for the modeling of monuments, as they combine the accuracy of the topographic mapping with the completeness and resolution of the photogrammetric mapping [33] (p. 80). They can actually be considered as advanced geodetic stations that can measure the direction of a fictional optical line, joining points on the surface of the monument to a reference point on the scanner, as well as estimating the distance between them. As a result, they can produce Cartesian coordinates automatically using the triangulation principle [34] (p. 96). Both airborne and terrestrial laser scanners can acquire a huge amount of 3D data very quickly, and the combination of the point clouds with high-resolution color digital images can achieve a higher level of detail together with good metric accuracy [35] (p. 1).

**Figure 10:**
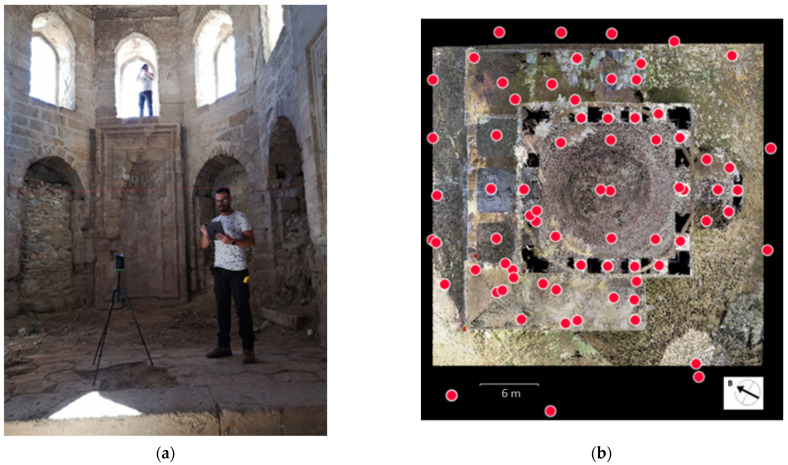
Terrestrial laser scanning: (**a**) data collection in the interior of the monument; (**b**) laser scanning positions indicated with red dots.

For the complete geometric documentation of the Mehmet Bey Mosque, a 3D laser scanning campaign was carried out using the BLK360 Imaging Laser Scanner, manufactured by Leica Geosystems in Heerbrugg, Switzerland, (Figure 10a), which was provided by the Department of Rural and Surveying Engineering of the Aristotle University of Thessalonikiand has the following characteristics [36]:

- 15 MP 3-camera system, 150 MP full dome capture, HDR, LED flash, calibrated spherical image, 360° × 300°;
- Longwave infrared camera, thermal panoramic image, 360° × 70°;
- Range: 0.6 to 60 m;
- Point measurement rate up to 360,000 pts/s;
- 3D point accuracy 6 mm at 10 m/8 mm at 20 m;
- Designed for indoor and outdoor use.

The monument was scanned from 24 internal and 46 external positions (Figure 10b) so that laser imaging for the whole building could be acquired. The recording of points on some surfaces with high elevation, such as the external shell of the central dome (approx. 25 m), was difficult, which necessitated their completion through photogrammetric methods. Indeed, one of the drawbacks of laser scanners is their limited range in the vertical direction, as they have to be placed at a great distance from the surface in case it extends to a great height [37] (p. 35). However, such solutions cannot always be applied because of restrictions imposed by the spatial or urban surroundings of the monument [38] (p. 1).

### 2.2. Data Processing

#### 2.2.1. Land Surveying Data

The processing of the topographic measurements initially involves solving the polygon traverses to determine the coordinates of their vertices and checking the closure errors. The calculations were carried out in Excel spreadsheets of the Department of Geodesy and Topography of AUTh, in which the X and Y coordinates of the 4 dependence and orientation points, the measured angles (β) and the horizontal distances (S) between the traverse stations were used as input data. Table 3 presents the number of vertices, the length, the angular error Wβ, the linear error δs and the maximum errors for each traverse. The maximum errors were calculated for primary traverses on flat ground and a scale of 1:500. The closure angular and linear errors were acceptable.

**Figure 11:**
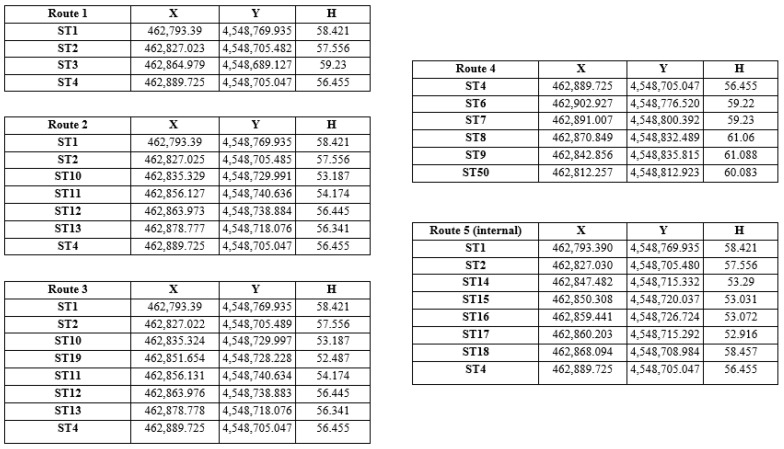
Calculated coordinates of the traverse vertices.

Subsequently, we calculated the coordinates of points of interest and control points in Excel spreadsheets of the Department of Geodesy and Topography, AUTh. As an input, we used the coordinates (X, Y, H) of the traverse stations and the orientation points (Figure 11), the height of the instrument, the target height for each point, the horizontal direction, the zenith angle and the slope distance.

**Figure 12:**
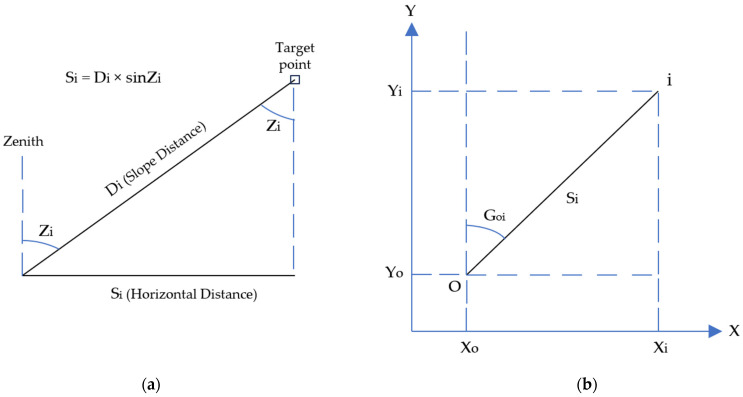
Spatial sketches of (**a**) the zenith angle Zi and (**b**) the direction angle G_oi_.

The horizontal coordinates (X, Y) and the elevation H of points of interest were calculated automatically based on the formulas of the first fundamental problem ((1) and (2)) and trigonometric leveling (3), respectively (Figure 12):

X_i_ = Χ_ο_ + D_i_ sin Z_i_ sin (G_oi_) (1)

Y_i_ = Y_o_ + D_i_ sin Z_i_ cos (G_oi_) (2)

H_i_ = H_o_ + D_i_ cos Z_i_ + H_j_ − H_τ_ (3)

- X_i_, Y_i_, H_i_: horizontal coordinates and elevation of the target point;
- X_ο_, Y_ο_, H_ο_: horizontal coordinates and elevation of the traverse station;
- D_i_: the slope distance between the target point and the mechanical center of the geodetic station;
- Z_i_: the zenith angle;
- G_oi_: the direction angle;
- H_j_: the height of the mechanical center of the geodetic station from the traverse station;
- H_τ_: the target height.

#### 2.2.2. Photogrammetric Data

Image-based modeling techniques use 2D image measurements (i.e., shading, texture, contours, edge gradients, etc.) and projective geometry through a perspective camera model. The generation of 3D models is achieved by applying dense stereo reconstruction algorithms on a set of unordered images that illustrate the object [39] (p. 12). This process can be described as an indirect derivation of geometries through stereoscopic or multiscopic restitution or bundle adjustment [40] (p. 1). Over 40 different types of open-source and commercial photogrammetric software are available for the generation of 3D models, all of which generally follow a five-step process [41] (p. 2):

1. Feature detection and matching;
2. Triangulation;
3. Dense point cloud generation;
4. Surface/mesh generation;
5. DSM and orthophoto generation.

The photogrammetric software that we used for the project is Agisoft Metashape Professional 1.7.0, which is suitable for close-range photogrammetric applications. The processing resulted in products such as the digital surface models (DSMs) and the orthophotomosaic of the historical complex, the 3D models of the mosque and the fountain, orthophotos of the facades, and horizontal and vertical sections.

**Figure 13:**
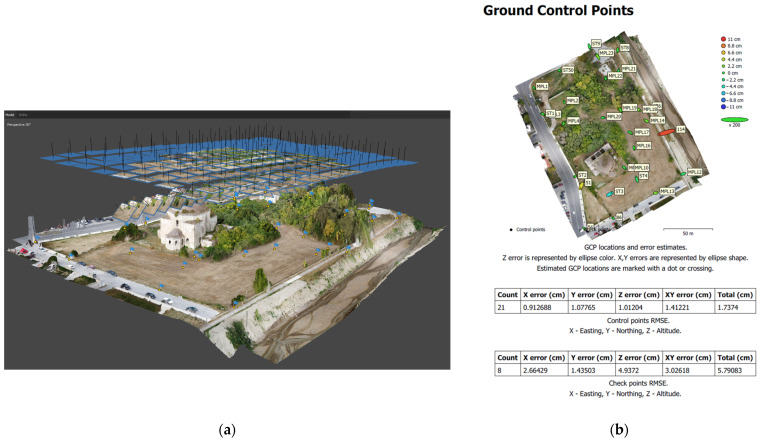
UAV image processing: (**a**) location of overlapping UAV images and GCPs; (**b**) GCP and CP error estimation in the processing report.

The bundle adjustment for the 3D representation of the complex began with the alignment, i.e., the relative orientation, of 182 aerial overlapping images (Figure 13a). Their absolute orientation, i.e., the georeference, was applied by introducing 29 GCPs in GGRS’87 as markers, with 8 of them being excluded from the bundle adjustment (check points). After the generation of a textured 3D mesh, a processing report about the CP accuracy, the camera parameters, etc., was exported. The control point accuracy was estimated at 1.7 cm, and the check point accuracy at 5.8 cm (Figure 13b). Afterward, the orthophotomosaic of the complex was generated with the single-image method of orthorectification.

**Figure 14:**
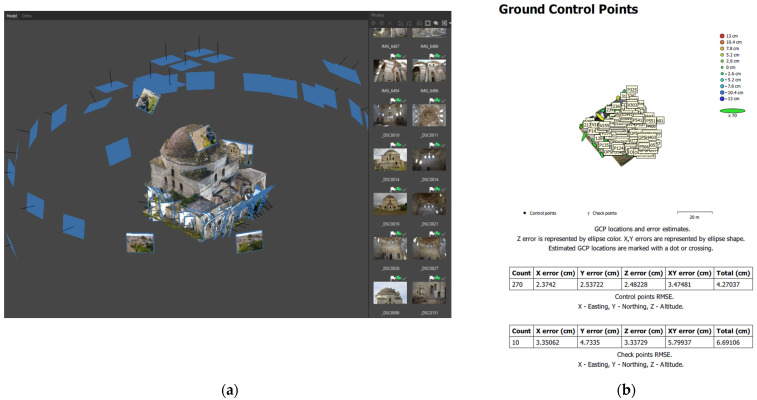
Combination of UAV and terrestrial images for the 3D modeling of the monument: (**a**) image processing; (**b**) GCP and CP error estimation in the processing report.

**Figure 15:**
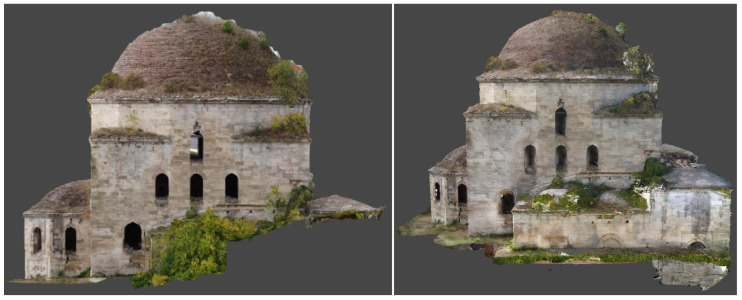
Completion of the eastern facade with terrestrial images.

For the 3D reconstruction of the mosque externally and internally, we used 140 overlapping images (Figure 14a). The aligning of the UAV images resulted in the formation of the external shell of the monument with gaps in the lower zones, due to the dense vegetation around the monument which limited visibility. These zones were completed with terrestrial images, which were successfully combined with the aerial ones using GCPs in an appropriate distribution (Figure 15). The terrestrial images also helped to improve the texture of the model, since the UAV images had a lower resolution.

The alignment and orientation of the images were carried out using a total of 280 markers in GGRS’87, 10 of which were defined as check points for accuracy control. The control points were automatically identified in all images, and their placement in the correct position was checked manually based on the facade sketches. The external and the internal shells of the monument were generated separately and then merged since they were both georeferenced in the same system.

In the processing report produced by the software, the average 3D absolute error of the model was estimated at 4.27 cm based on the control points and 6.69 cm based on the check points (Figure 14b). The generated orthophotos were used for the production of facade, vertical and horizontal section plans with a scale of 1:50 that required graphic accuracy of 1.5cm, with which the accuracy of the CPs should be compatible [42] (p. 7). According to the CP error estimation, the accuracy was approximately 3.0 to 5.5 cm lower than expected, possibly due to the use of different image scales for the completion of the mosque’s model. This accuracy would be more appropriate for plans with a scale of 1:200 and graphic precision of 6 cm.

**Figure 16:**
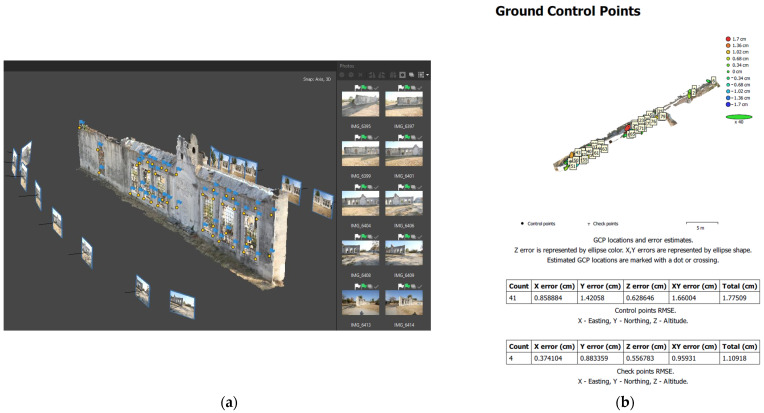
Three-dimensional modeling of the Ottoman fountain: (**a**) terrestrial image processing; (**b**) GCP and CP error estimation in the processing report.

The 3D model of the fountain (Figure 16a) was derived with the same process, using 18 terrestrial images, 41 control points and 4 check points, for which an accuracy of better than 2 cm was achieved (Figure 16b).

#### 2.2.3. TLS/Photogrammetric Data Fusion

Range and image-based techniques can both generate high-resolution 3D models, with range sensors capturing the geometry of the object thoroughly, in contrast to the image-based approach that represents just the main object structure. However, digital imagery can enhance the realistic perception of a range-based model and help fill any gaps with information on edges and linear surface features [43] (p. 1). For instance, a terrestrial laser scanner may not be able to capture a roof when the target building is not adjacent to taller buildings [44] (p. 37). For these reasons, in many cases, an optimal result with sufficient details is achieved with the fusion of these techniques [45] (p. 2).

**Figure 17:**
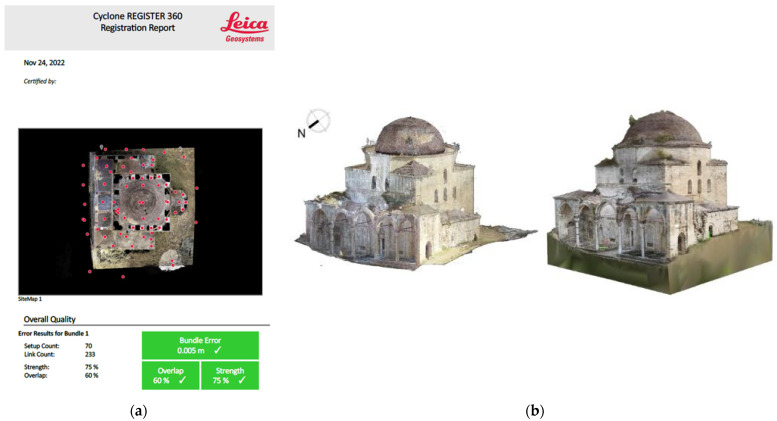
Processing of the TLS data: (**a**) report with an acceptable bundle error indicated with a checkmark; (**b**) TLS and photogrammetric data fusion.

In our case study, the combination of range and image-based data was the most successful 3D modeling method for the mosque. Initially, the registration of the laser scanning data was performed in the Leica Cyclone Register 360 PLUS (BLK Edition) software [46]. The point clouds were joined by the Cloud-to-Cloud method, which uses features and points in the overlap area of the scans for alignment. The bundle error was estimated at 5 mm (Figure 17a). The point clouds were further processed for the removal of noise and unnecessary or inaccurate points. In the mesh generation stage, other programs could have given quite different results for the same object [47] (p. 6).

After the scan registration, the 3D mesh of the mosque emerged with an incomplete form of the central dome, due to the technical difficulties created by the great height of the monument and the intense vegetation around it. This problem was confronted by integrating aerial and terrestrial imagery into the range-based mesh, which was implemented using the photogrammetric software RealityCapture 1.3 [48]. The two datasets were related into a common reference system using 7 control points measured by the total station survey, with a mean triangulation uncertainty of 2.1 cm and mean reprojection error of 0.57 pixels. This procedure achieved the completion of the dome structure, which was almost entirely absent from the laser scanning model [49] (p. 420). Thus, the final georeferenced 3D model acquired better visual quality and a more realistic texture (Figure 17b).

## 3. Results

The campaign resulted in the generation of a survey plan for the Mehmet Bey complex, along with useful photogrammetric products, such as a 3D model of the terrain, an orthophotomosaic, a DTM, a DSM and longitudinal sections. The monuments were documented with 3D models, facade orthophotos, orthorectified horizontal and vertical sections and, finally, with architectural plans.

### 3.1. Survey Plan

**Figure 18:**
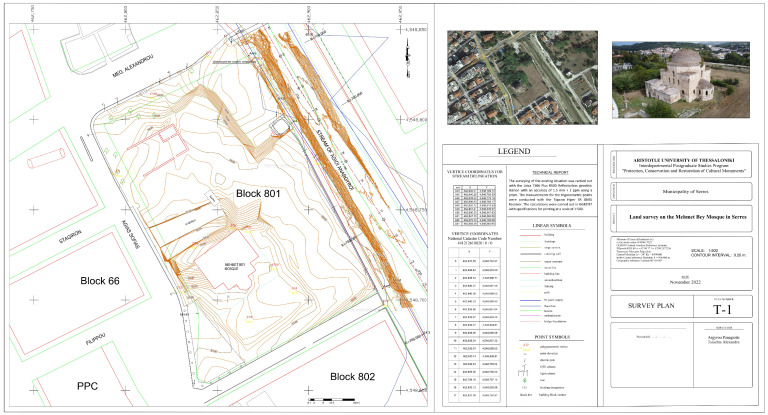
Survey plan of the historical complex.

One of the main products of our study was the survey plan of the historical block on a scale of 1:500, which was designed in Autodesk’s AutoCAD 2022 software based on the identified coordinates of the points of interest in GGRS’87 (Figure 18). The drawing was created with guidance from the field sketch and the orthophotomosaic of the study area that was generated from the photogrammetric processing of UAV images. The diagram illustrates the existing natural and artificial structures, such as the stream, roads, sidewalks, fences and buildings. The terrain relief was mapped with 0.20 m iso-elevation curves, which were generated in Autodesk’s Civil 3D 2022 Metric software, based on the identified elevation points and the points of interest. 

### 3.2. Photogrammetric Products

**Figure 19:**
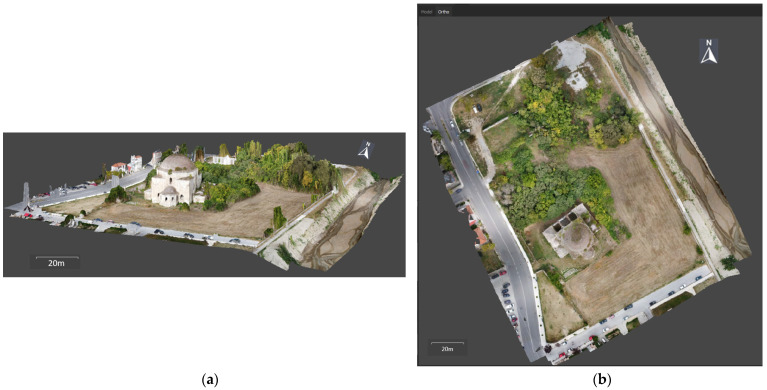
Products of the UAV image processing for the historical complex: (**a**) 3D photogrammetric model; (**b**) orthophotomosaic.

The photogrammetric processing resulted in the 3D modeling of the historical block (Figure 19a), the mosque and the fountain, from which products such as DSMs, orthophotos and sections were derived.

The orthophotomosaic of the study area was generated automatically, with the application of an orthorectification algorithm to the 3D model (Figure 19b). Orthorectification is a single-image method that, however, requires multi-image processing to determine the object’s relief.

**Figure 20:**
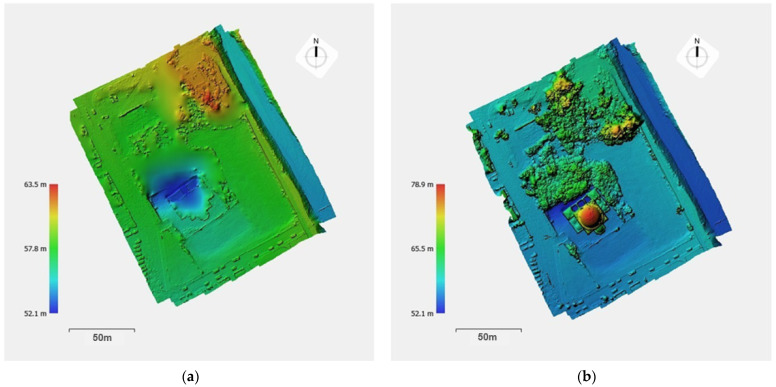
Altitude of the historical complex with a vertical scale in meters: (**a**) digital terrain model (DTM); (**b**) digital surface model (DSM).

**Figure 21:**
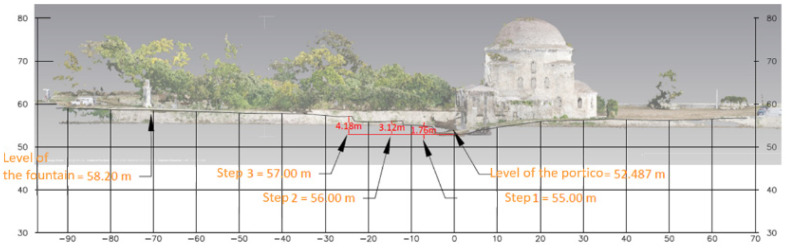
Longitudinal section on the N-S axis.

The digital surface model (DSM) and the digital terrain model (DTM) illustrate the relief of the study area rendered in color zones, corresponding to altitude values (Figure 20). The DTM was produced with the selection “Point Class: Ground” so that the elevations of the surfaces, such as the mosque or the vegetation, could be removed. In addition, longitudinal drawings on the north-southern and east-western axes were produced for the visualization of the terrain morphology, based on vertical sections of the 3D model (Figure 21).

**Figure 22:**
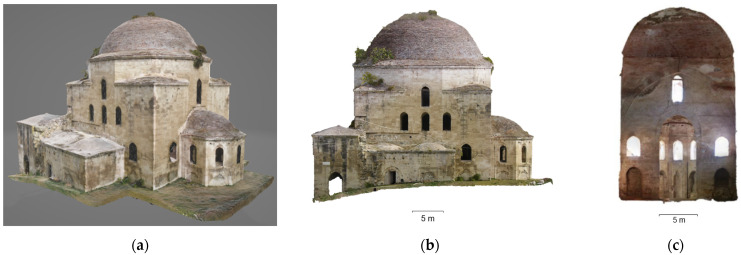
Photogrammetric products of the mosque: (**a**) 3D textured model (south-western view); (**b**) orthophoto of the western facade; (**c**) orthorectified interior section facing the south.

**Figure 23:**
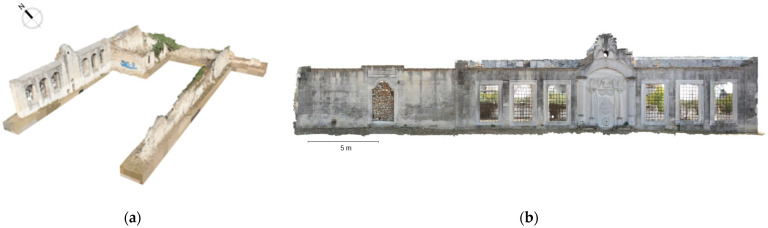
Photogrammetric products of the Ottoman fountain: (**a**) 3D model; (**b**) orthophoto of the northern facade.

The photogrammetric 3D model of the mosque was used for the production of orthophotos for the external facades and the internal sections (Figure 22). The interior lateral spaces were not incorporated into the photogrammetric model, as they were basically recorded through laser scanning. The 3D model of the Ismael Bey enclosure was used for the production of orthophotos of the fountain, the mausoleum and the perimetric walls (Figure 23).

### 3.3. TLS/Photogrammetric Products

**Figure 24:**
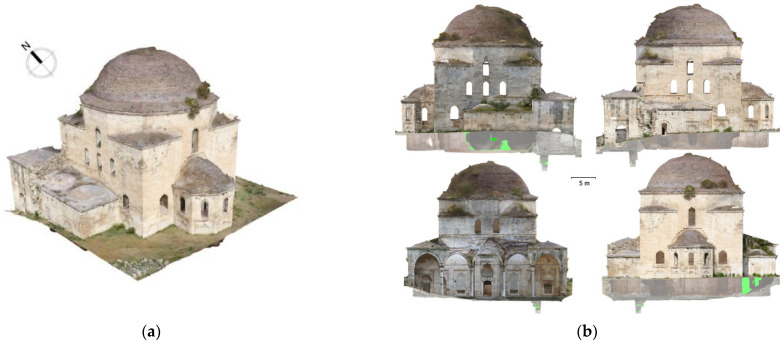
Products of the TLS–photogrammetric data fusion: (**a**) textured 3D model; (**b**) facade orthophotos (east, west, north, south).

**Figure 25:**
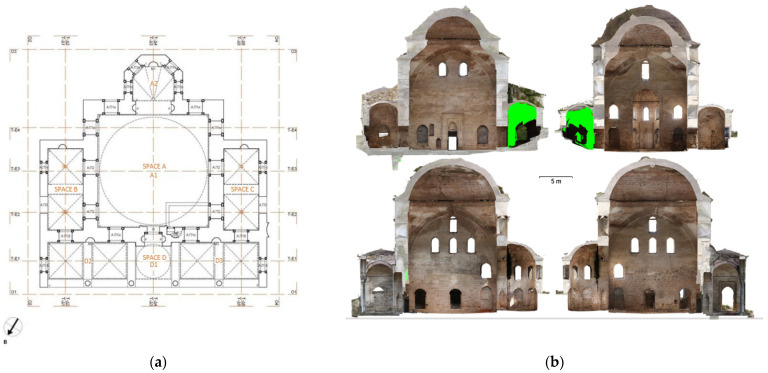
Vertical sections of the 3D model: (**a**) vertical section positions marked on a floor plan; (**b**) orthorectified vertical sections (north, south, east, west). The plan in Figure a was produced by the architecture team: Panagiotis Antonellos, Periklis Maniotis, Eleftherios Matzouneas, Emmanouil Nikiforos and Alexandros Hecht.

**Figure 26:**
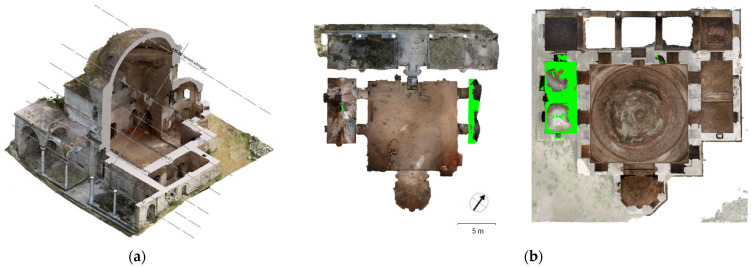
Horizontal sections of the 3D model: (**a**) horizontal section levels marked on an axonometric projection; (**b**) orthorectified horizontal sections in different levels.

**Figure 27:**
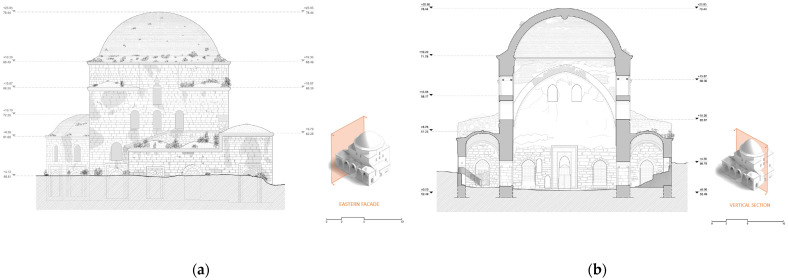
Architectural plans of: (**a**) the eastern facade and (**b**) the vertical section facing north. The plans were produced by the architecture team: Panagiotis Antonellos, Periklis Maniotis, Eleftherios Matzouneas, Emmanouil Nikiforos and Alexandros Hecht.

**Figure 28:**
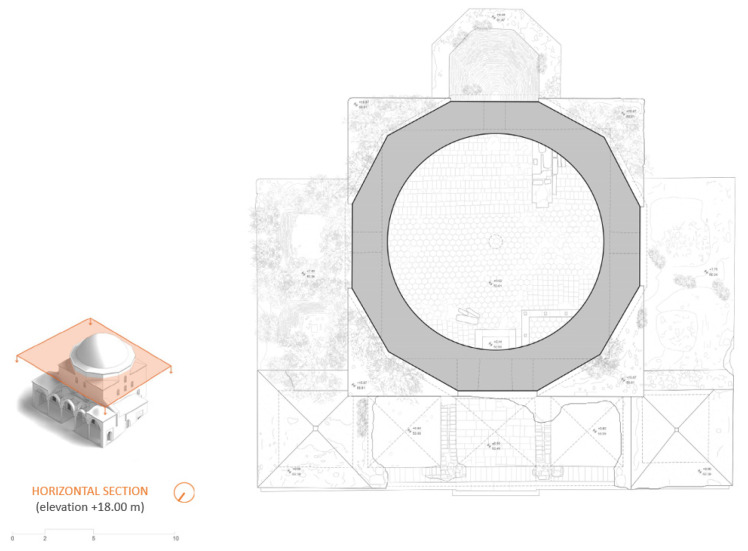
Architectural plan of a horizontal section, e.g., at an elevation of +18 m., produced by the architecture team: Panagiotis Antonellos, Periklis Maniotis, Eleftherios Matzouneas, Emmanouil Nikiforos and Alexandros Hecht.

The combination of TLS and photogrammetry resulted in a high-quality photorealistic model (Figure 24a), which provided a series of essential documentation products, such as the orthophotos of the external facades (Figure 24b), as well as vertical (Figure 25) and horizontal sections at desired levels (Figure 26) [50] (p. 86). These products served as a background for the generation of architectural plans (Figure 27 and Figure 28). The insufficient lighting in the eastern interior lateral space and the subsequent lack of coloring data caused a small area of green blur inside the model, as shown in the orthophotos. In other words, the geometry of this area has been recorded as a point cloud, but it has no texture assigned.

## 4. Accuracy Evaluation

The geometric documentation of the monument was carried out with caution at every stage, thus providing the desired products in two or three dimensions with satisfactory accuracy. According to the processing reports that were automatically produced by the software, the photogrammetric processing and the TLS/photogrammetry fusion resulted in an accuracy of 6 cm and 2.1 cm, respectively. We considered it important to proceed with further manual control of the orthophoto accuracy based on the accuracy of the total station survey. The errors of both the orthophotos from photogrammetry and TLS/photogrammetry fusion were graphically checked, with the points identified by the total station being considered as correct (ground truth) [51] (p. 434).

The checks were carried out in the Civil 3D 2022 Metric software using the line of best fit formula for a number of points of interest, on which a local 2D reference system was fitted for the rotation of the orthophotos. The deviation of all the characteristic points of interest from their actual position according to the topographic measurements was determined graphically on the orthophotos. Only the characteristic points that had not been used as control points during the bundle adjustment participated in this checking process. Afterward, we calculated the average of the errors, as well as the standard deviation.

**Figure 29:**
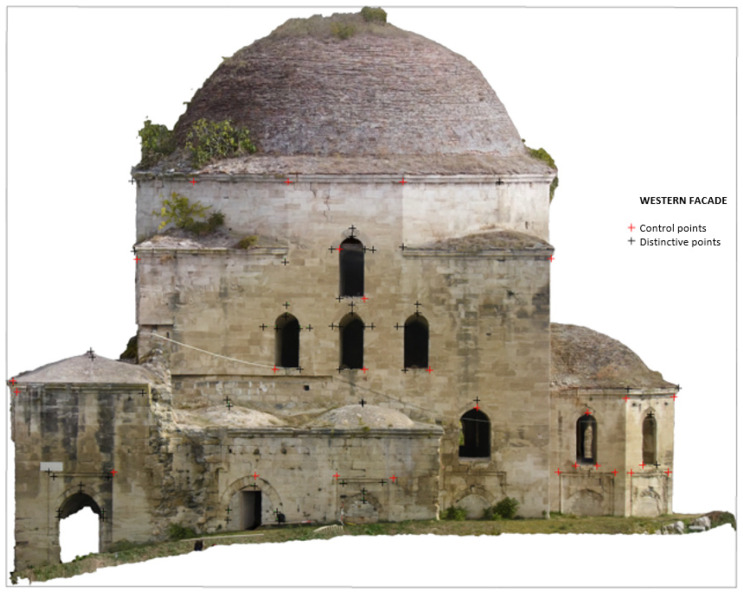
Error estimation for the western facade orthophoto derived from photogrammetric processing.

**Figure 30:**
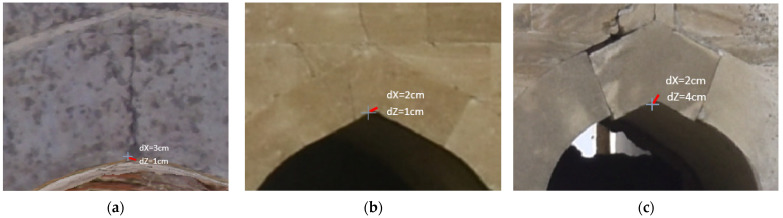
Representative examples of error estimation in close approximation for orthophotos derived from the photogrammetric model: (**a**) the vertical section facing the south (interior); (**b**) the southern facade; (**c**) the eastern facade.

**Figure 31:**
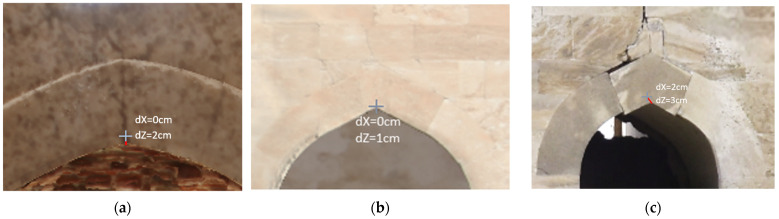
Representative examples of error estimation in close approximation for orthophotos derived from the TLS/photogrammetric model: (**a**) the vertical section facing the south (interior); (**b**) the southern facade; (**c**) the eastern facade.

Overall, satisfactory matching was found across the entire range of the orthophotos, even for out-of-plane points. Indicatively, checking the orthophoto of the western photogrammetric facade with a total of 48 points, an average error value of 3 cm was found, with a standard deviation of 4 mm along the *x* and *z* axes (Figure 29). The maximum error of the sample was determined at 6 cm, and the minimum at 1 cm. Similar error values were also found for the orthophoto of the western facade obtained from TLS/photogrammetric data fusion. In Figure 30, there are representative examples of error estimation for three characteristic points on orthophotos with a resolution of 3.5 mm/pix, derived from the photogrammetric model with 4.2 M triangles, while Figure 31 presents the same points on orthophotos with a resolution of 2 mm/pix, derived from the TLS/photogrammetric model with 1 B triangles. The texture of the orthophotos is natural. The deviation from the actual position is marked with a red line, and the error estimations dX and dZ are indicated with labels.

The results of the accuracies of the two applied modeling methods are presented in Table 4 and Table 5. The maximum error value is 6.6 cm, while the minimum is 1 cm, and the standard deviation ranges from 3 mm to 18 mm. Comparing the accuracy of the two methods, we could say that they present values in similar ranges, so we consider them to be equally reliable. The high error values of some orthophotos (4–6 cm) were most likely caused by the complex geometry of the monument, which necessitated the combination of many aerial and terrestrial images with different scales. The results of this checking process showed that the error estimation of the orthophotos lies between the accuracy of the two generated 3D models, so in fact, the TLS/photogrammetry fusion did not achieve significantly higher precision, as the automated report indicated.

## 5. Discussion

The geometric documentation of the case study was conducted with a variety of data acquisition methods, and the processing was completed with two different 3D modeling methods: aerial and terrestrial imagery fusion and the fusion of imagery with a laser point cloud. Table 6 is a summary of the photogrammetric and TLS data collection process regarding the equipment that was used, the number and size of the acquired images and laser scans, the surfaces that were recorded, and the recording height and position.

In addition, Table 7 presents comparative data about the two 3D modeling methods (processing software, registered images and laser scans, control points), the parameters of the final models and the products (triangles/vertices, quality level, texture resolution, accuracy estimation from software and manual checks). The photogrammetric 3D model is simpler with significantly less imaging information (140 aerial and terrestrial images) than the TLS/photogrammetric 3D model which contains 3429 images and 70 laser scans. In the first case, the photogrammetric model required a large number of control points in appropriate distribution so that the geometry of the building could be created with accuracy. In the second case, the large amount of data could easily be aligned by the software because of the high overlap and the geometric information that was provided by the laser scanning point cloud, so the use of only seven control points was sufficient. The texture quality is much higher in the interior of the second model, which contains a lot of curved surfaces and complex geometries, while the quality of the external shell is similar. The accuracy of the two models is also similar. Overall, the second processing method results in models of higher quality, but it definitely requires a more evolved computer system.

## 6. Conclusions

The implementation of new technologies for the geometric documentation of monumental buildings and complexes in the context of the protection of our cultural heritage is a constantly evolving subject of research. The case study of the Mehmet Bey Mosque has demonstrated how digital multi-source data acquisition techniques can be applied with the use of modern, high-precision sensors. The data collection process must be carefully planned and executed so that all the information is obtained with the best accuracy possible and any deficiency can be addressed during the processing, without the need to return to the study area.

In the case of hard-to-reach monuments of large dimensions, it seems that mapping is greatly facilitated by the use of UAVs, terrestrial laser scanners and geodetic stations with the ability to measure without a prism. In particular, the use of a terrestrial laser scanner enabled us to quickly and thoroughly capture a monument with complex geometry, both externally and internally. The scanned point cloud was supplemented with photogrammetric methods since it was not feasible for the scans to cover the entire surface for the creation of a complete 3D model. The upper part of the building, i.e., the central dome, had to be completed with UAV images, which necessitated the fusion of terrestrial images, as well, for the achievement of a photorealistic texture. The different image scales degraded the accuracy of the final model. Another issue that arose is the blurring of the range-based 3D model in the area of the eastern interior lateral space due to the poor lighting conditions during the scanning process. In spite of these problems, the outcome was satisfactory, and it further proved that these two technologies, when properly implemented, can supplement each other in generating high-quality textured 3D models of monumental buildings and architectural details.

The image-based 3D model proved to be in the same range of accuracy. However, a visual comparison between the two models makes it clear that laser scans offer more geometric detail and a significantly improved result on the curved interior surfaces compared to simple photogrammetric processing. Special care is needed when acquiring images and measuring control points in interior lateral spaces so that it is possible to connect them with the main structure for the creation of a complete photogrammetric model. In the case of the mosque, the lateral spaces were not included in the 3D photogrammetric model, as laser scanning was the only method chosen for their recording.

In conclusion, the recommended methodology for the 3D modeling of complex cultural heritage buildings and specific architectural details is the combination of laser scanning and photogrammetric data, which can result in high geometric accuracy and high-quality texture, respectively. Data collection with a UAV proves to be essential for monuments of large dimensions so that the external shell of the roof or the domes and the upper zones can be recorded with dense point clouds. In addition, the TLS data collection is found to be necessary for projects with similar specifications of resolution and accuracy. As a result, both of them have to be used for the monitoring of large buildings in high resolution. As for the recording of specific architectural details, photogrammetry can offer higher resolution, unless a triangulation laser scanner is used.

The set of the products that were generated in the case study with both methods (survey plan, DEMs, orthophotomosaic of the complex, longitudinal sections, 3D models, orthophotos of facades, sections) documented the monumental complex in its specific phase completely and, at the same time, served as a background for an extensive interdisciplinary study for the restoration of a valuable surviving sample of the Ottoman architecture in Greece.

## Figures and Tables

**Table 1:** DJI Mavic Air 2 drone quadcopter UAV parameters [26].

Model Name	DJI Mavic Air 2
Weight	570 g
Max Flight Time (without wind)	34 min
Max Flight Distance	18.5 km
Vertical Accuracy Range	±0.1 m (with vision positioning); ±0.5 m (with GPS positioning)
Horizontal Accuracy Range	±0.1 m (with vision positioning); ±1.5 m (with GPS positioning)
Satellite Systems	GPS + GLONASS
Sensor	1/2″ CMOS; effective pixels: 12 MP and 48 MP
Lens	FOV: 84°; equivalent focal length: 24 mm; Aperture: f/2.8; focus range: 1 m to ∞
Max Photo Resolution	48 MP 8000 × 6000 pixels

**Table 3:** Number of vertices (n), length (S), closure errors (W_β_, δ_s_) and error limits (W_β_, δ_s_ max) of the polygon traverses.

Traverses	Vertices n	Length S (m)	W_β_ (c)	W_β_ max (c)	δ_s_ (cm)	δ_s_ max (cm)
Traverse 1	4	143.47	2.76	4.00	1.79	10.99
Traverse 2	7	172.55	1.61	5.29	2.55	11.57
Traverse 3	8	178.80	1.13	5.66	3.91	11.68
Traverse 4	6	203.66	4.58	4.90	1.92	12.13
Traverse 5 (interior)	8	155.77	0.97	5.66	0.98	11.24

**Table 4:** Error estimation for the orthophotos derived from TLS/photogrammetric data fusion.

Laser Scanner–Photogrammetry Orthophotos	Mean (μ)		Standard Deviation (σ)	
	dx (cm)	dΖ (cm)	dX (cm)	dΖ (cm)
Eastern facade	6.6	2.8	1.2	0.5
Western facade	2.5	2.7	0.3	0.4
Northern facade	3.5	4.8	0.6	0.9
Southern facade	2.7	5.8	0.6	1.3
Interior—East	1.0	2.3	0.3	0.6
Interior—West	3.1	2.7	0.8	0.7
Interior—North	1.6	1.2	0.5	0.3
Interior—South	5.4	3.4	1.8	1.1

**Table 5:** Error estimation for the orthophotos derived from photogrammetric processing.

Photogrammetric Orthophotos	Mean (μ)		Standard Deviation (σ)	
	dX (cm)	dΖ (cm)	dX (cm)	dΖ (cm)
Eastern facade	6.2	3.0	1.2	0.6
Western facade	3.1	3.0	0.4	0.4
Northern facade	5.1	5.9	0.9	1.0
Southern facade	3.7	6.5	0.8	1.4
Interior—East	2.5	3.3	0.7	1.0
Interior—West	4.1	3.9	1.0	1.0
Interior—North	2.0	1.8	0.6	0.5
Interior—South	5.4	3.3	1.8	1.1

**Table 6:** Summary of the photogrammetric/TLS data collection process.

	Aerial Photogrammetry	Terrestrial Photogrammetry	Terrestrial Laser Scanning
Equipment	DJI Mavic Air 2—drone quadcopter UAV	Canon EOS 7D DSLR camera	Leica BLK360 Imaging Laser Scanner
Number of images/laser scans	255 images	3708 images	70 laser scans
Recorded surfaces	The Mehmet Bey block (184 images) and the mosque (71 images)	The mosque (3500 internal and external images); the fountain and the burial enclosure of Ismael Bey (208 images)	The mosque (24 internal and 46 external laser scans)
Recording height/position	Hovering height of 43m above the block and 18 m around the mosque	Rings of approx. 5 m and 10 m around the mosque, the fountain and the burial enclosure; on accessible open window frames of the mosque for the recording of the floor	Stations with point cloud overlap (approx. every 5 m); on accessible open window frames of the mosque for the recording of the upper internal zones at a height of 10 m
Time spent in the field	1 h	6 h	10 h
Data size	1 GB	80 GB	70 GB

**Table 7:** Comparative data about the workflows for the 3D modeling of the mosque.

	Photogrammetric Model	TLS/Photogrammetric Model
Processing software	Agisoft Metashape Professional 1.7.0	RealityCapture 1.3
Registered images/laser scans	140 images	3429 images; 70 laser scans
Control points	280	7
Triangles/vertices	4.2 M/2.1 M	Initial model: 1 B/0.5 B Simplified model: 5 M/2.5 M
Quality level	Medium	Normal
Texture resolution	4096 × 4096	16384 × 16384
Accuracy estimation from software	6.7 cm	2.1 cm
Accuracy estimation from manual checks on orthophotos (max. errors)	6.6 cm	6.5 cm
Products	Textured and georeferenced 3D model; facade orthophotos; horizontal and vertical sections	Textured and georeferenced 3D model; facade orthophotos; horizontal and vertical sections

## Data Availability

All data are available from the corresponding author upon reasonable request.
